# The COPII subunit MoSec24B is involved in development, pathogenicity and autophagy in the rice blast fungus

**DOI:** 10.3389/fpls.2022.1074107

**Published:** 2023-01-09

**Authors:** Hui Qian, Lixiao Sun, Minghua Wu, Wenhui Zhao, Mengyu Liu, Shuang Liang, Xueming Zhu, Lin Li, Zhenzhu Su, Jianping Lu, Fucheng Lin, Xiaohong Liu

**Affiliations:** ^1^ State Key Laboratory for Managing Biotic and Chemical Treats to the Quality and Safety of Agro-products, Institute of Biotechnology, Zhejiang University, Hangzhou, China; ^2^ State Key Laboratory for Managing Biotic and Chemical Treats to the Quality and Safety of Agro-products, Institute of Plant Protection and Microbiology, Zhejiang Academy of Agricultural Sciences, Hangzhou, China; ^3^ College of Life Sciences, Zhejiang University, Hangzhou, China

**Keywords:** COPII, Sec24, *Magnaporthe oryzae*, autophagy, pathogenicity, signaling pathway

## Abstract

The endoplasmic reticulum (ER) acts as the starting point of the secretory pathway, where approximately one-third of the proteins are correctly folded and modified, loaded into vesicles, and transported to the Golgi for further processing and modification. In this process, COPII vesicles are responsible for transporting cargo proteins from the ER to the Golgi. Here, we identified the inner shell subunit of COPII vesicles (MoSec24B) and explored the importance of MoSec24B in the rice blast fungus. The targeted disruption of MoSec24B led to decreased growth, reduced conidiation, restricted glycogen and lipids utilization, sensitivity to the cell wall and hypertonic stress, the failure of septin-mediated repolarization of appressorium, impaired appressorium turgor pressure, and decreased ability to infect, which resulted in reduced pathogenicity to the host plant. Furthermore, MoSec24B functions in the three mitogen-activated protein kinase (MAPK) signaling pathways by acting with MoMst50. Deletion of MoSec24B caused reduced lipidation of MoAtg8, accelerated degradation of exogenously introduced GFP-MoAtg8, and increased lipidation of MoAtg8 upon treatment with a late inhibitor of autophagy (BafA1), suggesting that MoSec24B regulates the fusion of late autophagosomes with vacuoles. Together, these results suggest that MoSec24B exerts a significant role in fungal development, the pathogenesis of filamentous fungi and autophagy.

## Introduction

Rice blast, a fungal disease caused by the filamentous ascomycete fungus *Magnaporthe oryzae*, represents a severe threat to global food production (Wilson and Talbot, 2009). When *M*. *oryzae* infects rice leaves, a round top-shaped appressorium forms, which is a special infection structure (Hamer et al., 1988; Talbot et al., 1993). Once the appressorium accumulates a high concentration of glycerol and generates a turgor pressure of up to 8.0 MPa, under the action of penetration peg, cellular turgor is translated into mechanical pressure that makes *M. oryzae* rupture the plant epidermis to produce invasive hyphae (IHs) (deJong et al., 1997; Thines et al., 2000; Fernandez and Orth, 2018). More and more evidence points to the fact that IHs entering plant cells secret effector proteins through two distinct secretion systems. Cytoplasmic effectors enter the host cell through a biotrophic interfacial complex (BIC)-dependent approach (Giraldo et al., 2013; Mochizuki et al., 2015). In contrast, apoplastic effectors are secreted via the traditional ER-Golgi secretion pathway (Giraldo et al., 2013; Mochizuki et al., 2015). Many proteins engaged in the traditional secretion pathway have been reported to regulate the secretion of apoplastic effector proteins, and the ER chaperone protein MoLhs1 plays an important role in modulating the secretion of MoSlp1 (Yi et al., 2009).

The basic coat protein complex II (COPII), which serves as a critical member of the traditional secretory process, primarily binds to cargo receptors and transports proteins synthesized in the ER to the Golgi (Novick et al., 1980; Otte and Barlowe, 2002). It acts as a highly conserved protein complex that ranges from fungi to mammals. The COPII complex comprises at least five cytosolic proteins: Sar1, Sec23, Sec24, Sec13, and Sec31. Sec23-Sec24 are for the inner COPII coat, and Sec13-Sec31 are for the outer (Bonifacino and Glick, 2004). Sec24, the subunit of COPII vesicles, is responsible for recruiting protein cargoes into nascent vesicles. Yeast has two Sec24 paralogs, Sfb2/Iss1 (56% identity) and Sfb3/Lst1 (23% identity), which interact with Sec23 to form the COPII-cargo adaptor complex. Mammals have four Sec24 paralogs: Sec24A, Sec24B, Sec24C and Sec24D (Tang et al., 1999; Cui et al., 2019). The diversity of Sec24 proteins is what allows COPII vesicles to package more cargo proteins, making the transport process more enriched (Miller et al., 2002). In *M*. *oryzae*, inner COPII coat MoSec24-2 can regulate the secretion of effector proteins together with the auxilin-like protein MoSwa2 to inhibit the immune response of rice (Liu et al., 2021). This suggests that the function of COPII components is closely related to the pathogenesis of *M. oryzae*.

Apart from serving in the secretory process by itself, the COPII complex has been reported to be involved in MAPK signaling pathway and autophagy (Hamasaki et al., 2003; Wang and Lucocq, 2007). Previous studies found that knockdown of ERK2, a component of MAPK cascade, in Hela cells resulted in a significant decrease in the number of α1-antitrypsin transported by COPII and the number of ER exit site (ERES) *in vivo* (Farhan et al., 2010). Furthermore, ERK2 could phosphorylate Sec16, a key protein residing in ERES, and promote COPII vesicle generation (Farhan et al., 2010). Subsequently, another study found that overexpression of the cellular kinase ERK7 reduced the localization of Sec16 in the ERES, leading to disrupted secretion (Zacharogianni et al., 2011). These findings reveal that the MAPK signaling pathway is closely related to the early COPII secretion pathway. In *M. oryzae*, The MAPK pathway, including Mst11-Mst7-Pmk1, Mck1-Mkk2-Mps1, and Ssk2-Pbs2-Osm1, has been widely described to be involved in the appressorium-mediated infection process (Li et al., 2017). The bridging protein MoMst50, a key component on the Pmk1 pathway, can crosstalk the other two MAPK pathways to serve different roles and thus regulate the pathogenicity of the rice blast fungus (Park et al., 2006). In the Δ*Momst50* mutant, the phosphorylation level of Pmk1 was significantly reduced, appressorium formation was greatly impaired, and the pathogenicity was almost completely lost (Li et al., 2017). When the homologous protein of Mst50 is disrupted in other pathogenic fungi such as *Ustilago maydis* (Mayorga and Gold, 2001), *Botrytis cinerea* (Schamber et al., 2010), and *Zymoseptoria tritici* (Kramer et al., 2009), the fungal development and pathogenicity are also impaired. Therefore, it is particularly interesting to further investigate how the COPII component and the MAPK pathway regulate the pathogenicity of *M. oryzae*.

Autophagy refers to the recruitment of Atgs (autophagy-associated proteins) to the autophagy initiation site (PAS) under stress conditions, initiating autophagy and forming autophagosomes with double-membraned structures. Then, autophagosomes fuse with vacuoles or lysosomes to degrade damaged or excess proteins in the cytoplasm and release energy, which is conducive to cell survival (Mizushima, 2007). Previous studies reported that mutants of the COPII coat proteins Sec23 and Sec24 and ERES proteins Sec12 and Sec16 in yeast were defective in autophagy (Ishihara et al., 2001). Further studies also showed that COPII vesicles provide a membrane source for the formation of autophagosomes (Shima et al., 2019), so COPII vesicles are closely related to autophagy. Autophagy has been proven to be involved in the process of development and pathogenicity in plant pathogenic fungi (Veneault-Fourrey et al., 2006). In *M. oryzae*, when ATG-related genes are disrupted, such as Atg1, Atg5, Atg8, the turgor pressure of appressorium is reduced and the pathogenicity is completely lost (Kershaw and Talbot, 2009). Although the existence of a link between COPII and autophagy, the specific mechanism by which COPII regulates autophagy in *M. oryzae* is not yet clear.

Here, we identified homologs of the COPII inner shell subunit Sec24 and obtained the null mutant of MoSec24B in *M*. *oryzae*. We found that MoSec24B, which is localized in the ER and Golgi, takes part in the spore production, appressorium maturation, glycogen and lipid droplets transport, and pathogenicity. MoSec24B works through the interaction with MoRas1 and MoMst50 to regulate the phosphorylation of Pmk1, Mps1, and Osm1 in response to stresses. In addition, MoSec24B regulates the pathogenicity of *M. oryzae* by participating in the fusion process of autophagosomes with vacuoles.

## Materials and methods

### Fungal strains, culture conditions, and quantitative RT-PCR

*M. oryzae* Guy11 was used as the wild-type strain (WT) in this study, and the Δ*Mosec24B* knockout mutant was generated from WT. All strains in this experiment were plated on complete medium (CM) and placed in an incubator at a temperature of 25 or 28°C, where the light-dark cycle was 16 h-light and 8 h-darkness (Beckerman and Ebbole, 1996). In the pharmacodynamic stress experiment, the strains were separately cultured on CM containing 100 μg/ml calcofluor white (CFW) (Yuanye Bio-Technology Co., Ltd, S26637), 0.004% sodium dodecyl sulfate (SDS) (Sangon Biotech, A100227-0500), 800 μg/ml Congo red (CR) (Sangon Biotech, A600324-0050), 0.6 M NaCl (HuShi, 10019318), 0.6 M KCl (Sangon Biotech, A100395-0500), 1.0 M sorbitol (Sangon Biotech, A100691-0500) for 8 days.

To detect the expression of sporulation-related genes in WT and Δ*Mosec24B*, cultured aerial mycelia were collected from cellophane, and total RNA was extracted and reverse transcribed into cDNA using a reverse transcription kit (TaKaRa, Japan). All primers were designed online by Integrated DNA Technologies and are shown in **Table S1**.

### Targeted gene deletion and the complement system

Targeted gene mutants were generated by homologous recombination based on the gene knockout system that was performed by Prof. Lu (Lu et al., 2014). To construct a knockout vector for MoSec24B, 1.5 kb upstream/downstream flanking sequences of the targeted gene were cloned from *M. oryzae* genomic DNA with specific primers sec24B up/down(F) and sec24B up/down(R). A hygromycin resistance gene (HPH) sequence of 1.3 kb in length was amplified in pCB1003 using the primer pair HPH-F/HPH-R in the same way as described above. Finally, the three fragments, sec24B upstream, downstream, and HPH, were ligated with the linearization vector pKO1B digested with *Hin*dIII/*Xba*I in the presence of a fusion enzyme (Vazyme Biotech Co., Ltd, C113-02). The constructed vector was transferred into WT using ATMT (*Agrobacterium tumefaciens*-mediated transformation) (Vijn and Govers, 2003), and the targeted gene was replaced with HPH. Whether a selectable marker gene HPH in a genomic mutant is a single copy needs to be confirmed by qPCR. In general, when a genomic β-tubulin gene that is a single copy is used as a positive control, the mutant is considered to be a single copy gene that contains HPH resistance if the calculated copy number is 0.8-1.2 (Lu et al., 2014). For complementation experiments, the full-length DNA of MoSec24B without a terminator codon (TAA) was fused to the linearized vector pKD5 digested with *Bam*HI/*Sma*I. The constructed complementation vector was transferred into the gene knockout mutant Δ*Mosec24B* via ATMT, and the correct transformants were identified (Zhu et al., 2021). All primers mentioned above are in **Table S1**.

### Observation of cellular localization in *M. oryzae*

The pKD3-MoSec24B-mCherry was constructed by inserting a fragment of MoSec24B genomic DNA without a terminator codon (TAA) into a linearized vector pKD3 digested with XbaI (Li et al., 2012b). The constructed vector was transferred into WT by ATMT. To determine the cellular localization, an ER-located marker and Golgi-located marker called Slp1-GFP and Sft2-GFP were transformed into the strain bearing the MoSec24B-mCherry tag, respectively. Green and red fluorescence were observed in conidia with a confocal fluorescence microscope (Fv3000, 60 × oil). To further confirm the function of MoSec24B in transport between the ER and the Golgi, we treated the conidia of the targeted strains with BFA (Sigma-Aldrich, B5936, 10 µg/ml) for 10 minutes. And co-localization was observed with a confocal fluorescence microscope using 0.1% dimethyl sulfoxide (DMSO) as a negative control. All primers mentioned above are in **Table S1**.

### Phenotypic assays

In the measurement of fungal growth experiments, WT, Δ*Mosec24B*, and Δ*Mosec24B::MoSEC24B* strains were grown on 7 cm plates with quantitative CM solid medium. The growth diameters were measured separately after 8 days. To evaluate spore production, we rinsed the spores of strains cultured for 11 days on 9 cm plates with 4 ml of sterile water, filtered them through three layers of filter paper, and counted the spores in the filtrate with a hemocytometer under a microscope. Conidial germination, appressorium formation, and cell collapse assays were performed as described by Qu et al. (Qu et al., 2020). To investigate the development of conidiophores, we first washed off the aerial hyphae growing on CM plates with sterile water and dried them, then cut the vegetative hyphae into long thin slices and finally placed them in a wet box for 24 h (Cao et al., 2016).

To test the pathogenicity of Δ*Mosec24B* on detached leaves, we inoculated mycelial plugs of WT, Δ*Mosec24B*, and Δ*Mosec24B::MoSEC24B* strains on detached 7-day-old barley (*Hordeum vulgare zj-8*) and 14-day-old rice (*Oryza sativa cultivar* CO39) leaves, and lesion areas were observed after 4 days. In the assay to detect the pathogenicity of Δ*Mosec24B* on rice seedlings, 1.5 ml of conidial suspension (1 × 10^5^ conidia/ml) was mixed with an equal volume of 0.4% (w/v) gelatin and sprayed uniformly on rice seedlings. The disease spot areas of each leaf were counted after 5–6 days. For penetration and invasive growth assays, we removed 20 µl of diluted conidial suspension (5 × 10^4^ conidia/ml) from WT, Δ*Mosec24B*, and Δ*Mosec24B::MoSEC24B* strains onto detached barley leaves and placed them at 25°C for 48 h. After incubation, the leaves were collected into 1.5 ml EP tubes, decolorized with methanol, and counted with a white light microscope (Kim et al., 2005).

### Staining of glycogen and lipid droplets

Conidial suspensions (20 µl) of WT, Δ*Mosec24B*, and Δ*Mosec24B::MoSEC24B* strains were taken out and dropped on artificial hydrophobic membranes to induce the formation of appressorium and incubated in a dark incubator at a temperature of 25°C. At 8 h, 16 h, and 24 h, respectively, the appropriate amount of spore solution was aspirated, and an equal volume of I_2_/KI solution was dropped on to stain the glycogen. The transport and degradation of glycogen were observed under a white light microscope. Similarly, lipid droplets were stained with Bodipy (Invitrogen, D-3922) solution, and the transport and degradation of lipid droplets were observed under a fluorescent microscope (Wei et al., 2020). For better staining of appressorium, 1 µl tricyclazole (10 µg/ml) (Macklin, T819453), which inhibits the formation of melanin in the cell wall of appressorium, was added to 1 ml conidial suspension (1 × 10^5^ conidia/ml).

### Mass spectrometry analysis

To identify the potential mechanism of MoSec24B regulation, we transformed the constructed MoSec24B-GFP vector into the Δ*Mosec24B* mutant by ATMT and screened to obtain the target strain. We cultured the target strain in CM liquid medium for two days and then extracted the total protein with protein lysis solution (50 mM Tris-HCl {Sangon Biotech, A600194-0005}, pH 7.4, with 1 mM EDTA {Sangon Biotech, A600436-0500}, 150 mM NaCl, and 1% Triton X-100{Sangon Biotech, A110694-0100}). The extracted protein supernatant was transferred to anti-GFP affinity beads and co-incubated for 4 h at 4°C, followed by four times washes with low salt buffer (50 mM Tris-HCl and 150 mM NaCl, pH 7.5), and finally 100 ul of 0.3 M glycine buffer ({Sangon Biotech, A610235-0005}, pH 2.5-3.0) was added and co-incubated with the affinity beads at 4°C for 15 minutes, and the supernatant obtained after centrifugation was stored at -80°C. In order to further identify the MoSec24B-related proteins, the LC-MS/MS data were performed by QE mass spectrometer (Shanghai Applied protein technology), and MASCOT was used to obtain information on the target protein peptide molecules (Zhu et al., 2021).

### Phosphorylation assays

To detect the phosphorylation levels of Pmk1 and Mps1, the wild-type and mutant strains were cultivated in 5 × YEG liquid medium for 2 days, and the proteins were extracted by TCA-acetone precipitation as described by Qu et al. (Qu et al., 2021). In addition, to further determine the phosphorylation levels of Mps1 under stress, the wild-type and mutant strains were cultured in 5 × YEG liquid medium for two days and induced with 100 µg/ml CFW for another hour. Then, the prepared protein samples were separated on 12.5% SDS-polyacrylamide gels and transferred to the PVDF membrane after electrophoresis. Finally, the analysis was performed by immunoblotting with anti-Phospho-p44/42 MAPK (1:500) (Cell Signal Technology, 4370), anti-p44/42 (1:500) (Cell Signal Technology, 9102), and anti-ERK1/2 MAPK (1:500) (Santa Cruz Biotechnology, sc-514302) antibodies.

For the phosphorylation analysis of Osm1, the wild-type and mutant strains were first incubated with CM liquid medium for two days and then induced with CM liquid medium supplemented with 0.6 M NaCl for 30 min, 60 min, and 90 min. To prepare the protein samples as described above, the analysis was performed by immunoblotting with anti-Phospho-p38 MAP kinase (1:500) (Cell Signal Technology, 9211) and anti-GAPDH (1:2000) (HuaBio, R1208-3) antibodies.

### Autophagy analysis

To detect the lipidized form of Atg8 during autophagy induction, WT and the Δ*Mosec24B* mutant strains were incubated in liquid CM medium for 36-40 h and then transferred to nitrogen-deficient medium SD-N for 2 h and 4 h. In addition, mycelium was treated with 500 nmol BafA1 (MCE, HY-100558), an agent that inhibits autophagosomes and vacuoles fusion, using 0.1% DMSO as a negative control. Mycelial proteins collected at different time points were extracted by the TCA-acetone method and detected with anti-ATG8 (1:2000, MBL, PM090) and anti-GAPDH antibodies.

To detect autophagic flux, the GFP-MoAtg8 vector already constructed in the laboratory was transferred into WT and Δ*Mosec24B* by ATMT, respectively (Liu et al., 2015). The strains were inoculated into CM and cultured for 7 days. The appropriate amount of mycelia was added to 150 ml of liquid CM medium and incubated for 36-48 h before being transferred to SD-N medium to be induced for 2 and 4 h. Identifying whether the protein loading of each strain is equal according to the protein GAPDH. After western blotting, the gray values of GFP-MoAtg8 fusion bands and free GFP bands were calculated with Image J.

### Yeast-two-hybrid, pull-down, and co-immunoprecipitation (co-IP) assays

The strain used for the yeast-two-hybrid system in this work was Y2HGold, and pGADT7-T and pGBKT7-53 were used as positive controls. The proteins were constructed onto the prey vector pGADT7 and the bait vector pGBKT7. The successfully constructed vectors were co-transformed into yeast receptor cells and cultured on SD-Leu-Trp and SD-Leu-Trp-Ade-His media.

The plasmids of GST, GST-MoSec24B, His-MoRas1, His-MoSep4, and His-Mst50 were transformed into *E*. *coli* strain BL21 (DE3) cells. The cells were lysed with cell lysis solution and the supernatant expressing GST protein was co-incubated with 60 μl glutathione agarose beads at 4°C for 2 h. After that, the agarose beads were washed five times with wash buffer, and finally, the supernatant expressing His protein was co-incubated with the agarose beads with GST tags for 2 h. The proteins were eluted down with elution buffer and detected by immunoblotting with anti-GST (1:2000) (Huabio, EM80701) and anti-His (1:2000) (Huabio, R1207-2) antibodies.

Firstly, the constructed empty GFP, MoRas1-GFP, MoSep4-GFP, and MoMst50-GFP vectors with glufosinate (BAR) resistance were cotransformed into the wild-type strain by ATMT with the MoSec24B-3×FLAG vector with G418 sulfate resistance, respectively. Transformants with both Bar and G418 resistance were screened by western blotting analysis. Next, total proteins of the obtained positive transformants were extracted by protein lysis solution and co-incubated with anti-GFP affinity beads for 4 h at 4°C, after which the affinity beads were washed four times with low salt buffer. Finally, after elution of the proteins with acidic glycine, the proteins were detected by immunoblotting with anti-GFP (1:5000) (Abcam, ab32146) and anti-Flag (1:2000) (Huabio, M1403-2) antibodies. All primers mentioned above are in **Table S1**.

## Results

### Identification of MoSec24 in *M. oryzae* and interactions within the COPII complex

**Figure 1:**
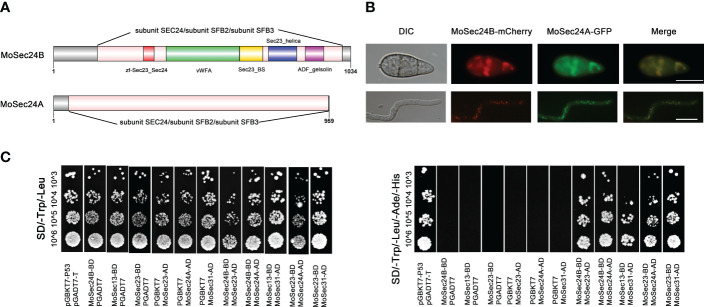
Identification of MoSec24 in *M. oryzae* and interactions within the COPII complex. **(A)** Structural domain analysis of the Sec24 homologous gene in *M. oryzae*. **(B)** Co-localization of MoSec24A-GFP and MoSec24B-mCherry in conidia and mycelium observed by fluorescence microscopy images. Scale Bar = 10 μm. **(C)** Analysis of the interactions between different subunits within the COPII complex by yeast-two-hybrid.

COPII can anchor the cytosolic domains of ER transmembrane proteins at specific times and wrap them to form vesicles that then shuttle from the ER to the Golgi apparatus (Gomez-Navarro and Miller, 2016). In turn, Sec24 plays a key role in this transport process as a cargo adapter for COPII that can bind to Sec23. In yeast, two homologues of Sec24 are Iss1/Sfb2 and Lst1/Sfb3. We queried the NCBI database with the protein sequences of these three types of Sec24 and identified two types of Sec24 (MGG_06569 and MGG_09564) in *M. oryzae*. MGG_06569 has 27.66%, 26.58%, 30.46% identity to Sec24, Iss1/Sfb2 and Lst1/Sfb3 in yeast respectively and MGG_09564 has 48.03%, 46.68%, 24.44% identity respectively. Therefore, we named MGG_09564 as MoSec24A and MGG_06569 as MoSec24B. Structural domain analysis through the NCBI database revealed that MoSec24A and MoSec24B share the common structural domain of subunit SEC24/subunit SFB2/subunit SFB3 (**Figure 1A**). We then looked at the co-localization of these two proteins and found that MoSec24B-mCherry and MoSec24A-GFP mostly co-located during the spore and mycelial stages (**Figure 1B**).

The COPII complex in yeast mainly picks cargo proteins through recognition sites on the inner shell subunit Sec23-Sec24, and then Sec23 recruits the outer shell subunit Sec13 by binding to Sec31 to form the final vesicle. We then first identified three other genes in COPII by protein sequence alignment, named MoSec23 (MGG_06910), MoSec13 (MGG_14666) and MoSec31 (MGG_01108). To further explore the interactions in COPII, we found that MoSec24A and MoSec24B, MoSec23 and MoSec24A, MoSec23 and MoSec24B, MoSec23 and MoSec31, and MoSec13 and MoSec31 interacted with each other by yeast-two-hybrid assay (**Figure 1C**), and these results are consistent with the findings in yeast.

### Subcellular localization of MoSec24B in *M. oryzae*

In order to characterize the function of Sec24 in *M. oryzae*, we knocked out MoSec24A and MoSec24B by employing the technique of MoSec24A and MoSec24B coding regions replacement with the hygromycin resistance gene (HPH). However, we only obtained the deletion mutant Δ*Mosec24B* (**Figure S1**).

**Figure 2:**
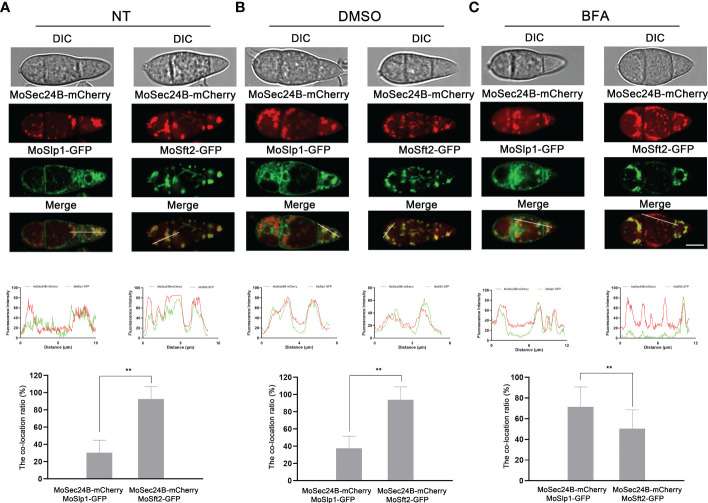
Subcellular localization of MoSec24B in *M. oryzae*. **(A)** Confocal fluorescence microscopy images observed the co-localization of MoSec24B-mCherry with ER-localized marker MoSlp1-GFP and Golgi-localized marker MoSft2-GFP, respectively, in untreated conidia (Fv3000, 60 × oil). **(B)** Confocal fluorescence microscopy images observed the co-localization of MoSec24B-mCherry with ER-localized marker MoSlp1-GFP and Golgi-localized marker MoSft2-GFP, respectively, in 0.1% DMSO-treated conidia (Fv3000, 60 × oil). **(C)** Confocal fluorescence microscopy images observed the co-localization of MoSec24B-mCherry with ER-localized marker MoSlp1-GFP and Golgi-localized marker MoSft2-GFP, respectively, in BFA-treated conidia (Fv3000, 60 × oil). Scale Bar = 5 μm. Fifteen spores were selected under each treatment (Normal, DMSO, BFA), the reticular or spot-like structures representing the ER and Golgi in each spore were labeled as number A, and the MoSec24-mCherry, which co-located with green fluorescence in each spore, was labeled as number B. (B/A) *100 was calculated as the co-localization percentage. The bar graphs indicate the percentage of localization of MoSec24B-mCherry in ER and Golgi, respectively, under different treatment conditions. Error bars represent the standard deviation. An analysis of the data was carried out using an unpaired two-tailed Student’s t-test. Asterisks represent statistically significant differences in the data (**P < 0.01).

To observe the subcellular localization of MoSec24B, we constructed three fluorescent fusion protein expression vectors (MoSec24B-mCherry, MoSlp1-GFP, and MoSft2-GFP). Slp1 and Sft2 are ER- and Golgi-located markers, respectively (Zhang et al., 2019; Qu et al., 2020). Then, MoSec24B-mCherry was co-transferred into WT with MoSlp1-GFP, and MoSft2-GFP vectors, respectively. When dormant conidia were observed by fluorescence microscopy, we found that only 30% of MoSec24B-mCherry co-localized with ER after untreated or treated with 0.1% DMSO, while 90% of MoSec24B-mCherry co-localized with Golgi (**Figures 2A, B**). When treated with Brefeldin A (BFA), a drug that inhibits the translocation of COPII vesicles from the ER to the Golgi, the co-localization of MoSec24B-mCherry with ER increased to 70%, and co-localization with Golgi decreased to 50% (**Figure 2C**). These results suggest that MoSec24B functions in the ER to the Golgi transport pathway.

### MoSec24B is vital for vegetative growth and conidiation

**Figure 3:**
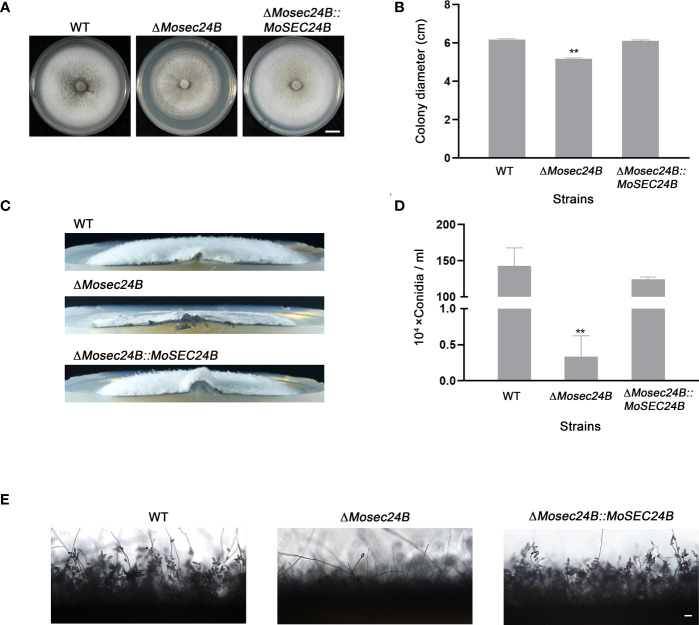
MoSec24B is involved in growth and conidiation. **(A)** WT, Δ*Mosec24B*, and Δ*Mosec24B::MoSEC24B* strains were cultured on CM solid medium for 8 days. Scale Bar = 1 cm. **(B)** Mycelial diameters of WT, Δ*Mosec24B*, and Δ*Mosec24B::MoSEC24B* strains. **(C)** Colony side views of aerial hyphae growth in WT, Δ*Mosec24B*, and Δ*Mosec24B::MoSEC24B* strains. **(D)** Conidiation of WT, Δ*Mosec24B*, and Δ*Mosec24B::MoSEC24B* strains. **(E)** Conidiophores of WT, Δ*Mosec24B*, and Δ*Mosec24B::MoSEC24B* strains were observed under a light microscope. Scale Bar = 50 μm. Error bars represent the standard deviation. An analysis of the data was carried out using an unpaired two-tailed Student’s t-test. Asterisks represent statistically significant differences in the data (**P < 0.01).

To further explore the basic biological functions of MoSec24B, growth and spore production assays were carried out. Compared to WT and the complementation strain, the Δ*Mosec24B* mutant exhibited slower mycelial growth and sparse aerial hyphae after 8 days of incubation on complete medium (CM) (**Figures 3A–C**). By statistical analysis of spore production, the number of conidia in Δ*Mosec24B* decreased sharply, with an average decrease of 427.5-fold compared to WT (**Figure 3D**). Moreover, the number of conidia in a conidiophore in the Δ*Mosec24B* mutant was also lower than that in WT (**Figure 3E**). Thus, we examined the expression of conidiation-related genes (*MoHOX2*, *MoCoNx2*, *MoFLBC*, *MoCNF1*, *MoGTA1*, and *MoMSTU1*) in the wild-type and mutant aerial mycelium and found that *MoCNF1*, which negatively regulates sporulation, was upregulated 1.5-fold in Δ*Mosec24B* (**Figure S2**). To analyze whether conidial germination and appressorium formation are normal in Δ*Mosec24B*, we induced conidia formation on artificial hydrophobic membranes and found that conidia of WT, Δ*Mosec24B*, and Δ*Mosec24B::MoSEC24B* strains could normally germinate and produce appressoria (**Figure S3**). These results above imply that MoSec24B plays a role in vegetative growth and conidiation but not in conidial germination and appressorium formation.

### MoSec24B is involved in pathogenicity

**Figure 4:**
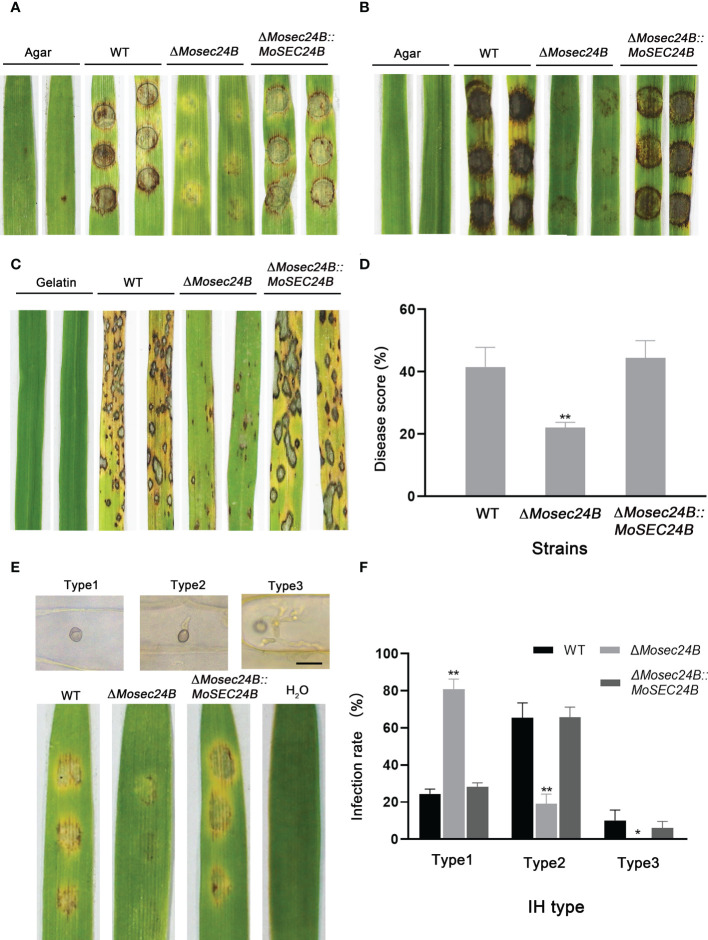
MoSec24B is important for pathogenicity. **(A)** Mycelial plugs from WT, Δ*Mosec24B*, and Δ*Mosec24B::MoSEC24B* strains were inoculated on detached barley leaves. **(B)** Mycelial plugs from WT, Δ*Mosec24B*, and Δ*Mosec24B::MoSEC24B* strains were inoculated on detached rice leaves. **(C)** Conidial suspensions (5 × 10^4^ conidia/ml) from WT, Δ*Mosec24B*, and Δ*Mosec24B::MoSEC24B* strains were sprayed on rice seedlings. **(D)** Disease areas of rice leaves whose length is 5 cm were measured using Photoshop CS6. **(E)** Conidial suspensions (5 × 10^4^ conidia/ml) from WT, Δ*Mosec24B*, and Δ*Mosec24B::MoSEC24B* strains were sprayed on barley leaves for 4 days, and IHs on barley cells were divided into three types. Scale Bar = 20 μm. **(F)** Statistical analysis of three IH types from WT, Δ*Mosec24B*, and Δ*Mosec24B::MoSEC24B* strains. Error bars represent the standard deviation. An analysis of the data was carried out using an unpaired two-tailed Student’s t-test. Asterisks represent statistically significant differences in the data (**P < 0.01, *P < 0.05).

To investigate whether MoSec24B functions in the pathogenicity of *M. oryzae*, the pathogenicity of mycelial plugs in barley and rice leaves was detected. Mycelial plugs of WT, Δ*Mosec24B*, and Δ*Mosec24B::MoSEC24B* strains were inoculated on detached barley leaves for four days. We observed that the disease spot formed by Δ*Mosec24B* was not extended and was significantly weaker than that of WT after 4 days (**Figure 4A**). Likewise, the results were similar in rice leaves (**Figure 4B**). A conidial suspension of 1 × 10^5^ conidia/ml was mixed with gelatin, sprayed on rice seedlings, and we observed only some small brown necrotic spots on rice seedlings leaves inoculated with Δ*Mosec24B*, while WT and Δ*Mosec24B::MoSEC24B* strains produced numerous and abundant typical spindle-shaped spots (**Figure 4C**). Lesion areas caused by Δ*Mosec24B* were significantly smaller than those caused by WT and Δ*Mosec24B::MoSEC24B* (**Figure 4D**). These results indicate that the deletion of MoSec24B results in reduced pathogenicity of *M. oryzae.*

To further analyze how the pathogenicity of Δ*Mosec24B* was reduced, we performed infection assays on barley leaves with a suspension of 5 × 10^4^ conidia/ml. Invasive hyphae (IHs) for all strains at 48 hours post-inoculation (hpi) were rated as from Type 1 to Type 3: Type 1, no penetration; Type 2, single or two short branches in only one cell; and Type 3, numerous branches in many other cells (**Figure 4E**). A statistical analysis of the types of IHs showed that only nearly 30% of IHs in the wild-type and complementation strains were Type 1, and nearly 80% of IHs in Δ*Mosec24B* were Type 1(**Figure 4F**). In addition, in the wild-type and complementation strains, 10% of IHs were Type 3, and almost no Type 3 IHs in Δ*Mosec24B*. These results indicate that the deletion of MoSec24B results in impaired appressorium-mediated infection and invasive hyphal growth.

### MoSec24B is involved in appressorium turgor and the mobilization of glycogen and lipid droplets

**Figure 5:**
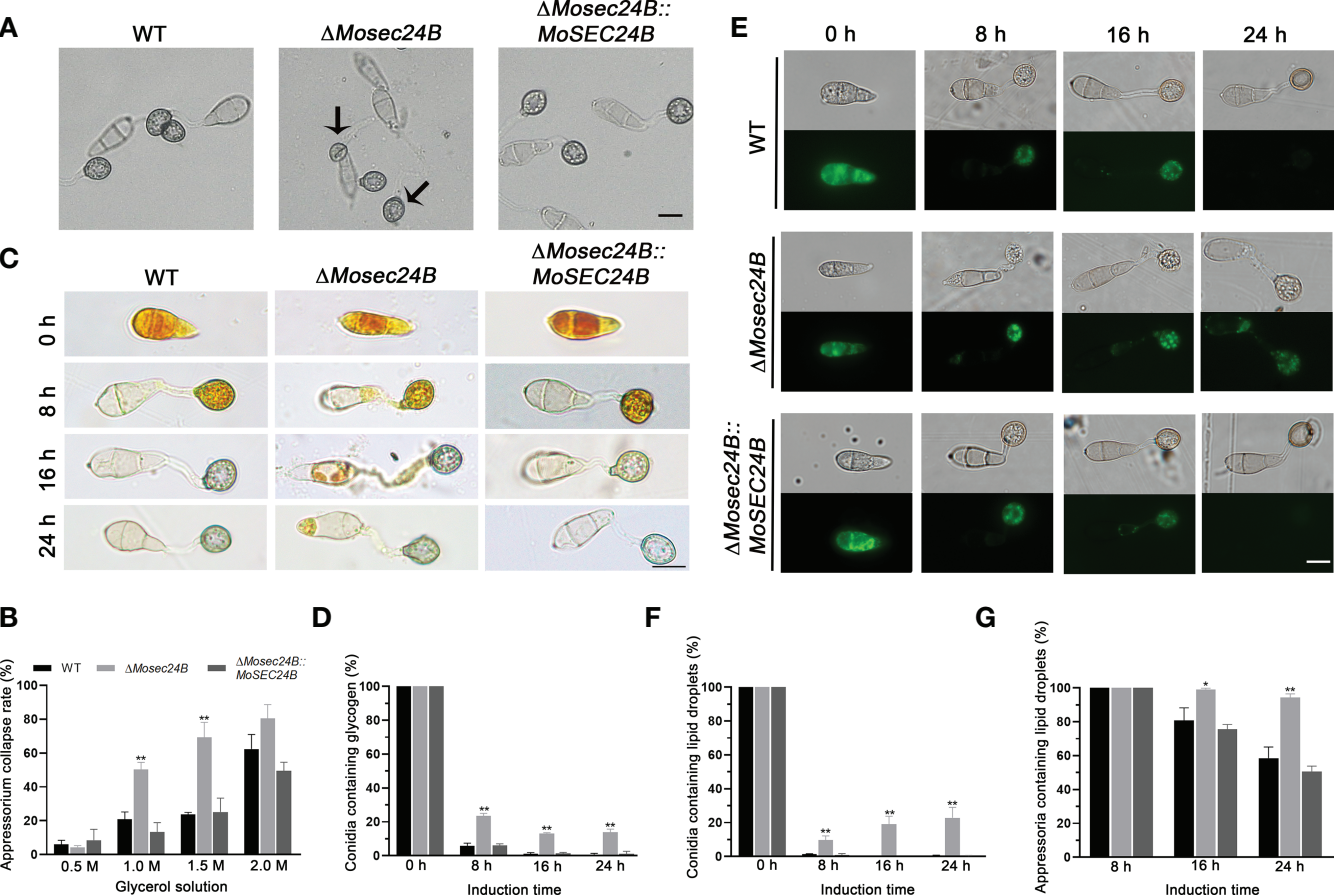
MoSec24B affects appressorium turgor and the mobilization of glycogen and lipid droplets. **(A)** Treatment with 1.0 M glycerol for appressoria collapse. Scale Bar = 10 μm. **(B)** Appressorium collapse rates of WT, Δ*Mosec24B*, and Δ*Mosec24B::MoSEC24B* strains under 0.5–2.0 M glycerol treatment. **(C)** Cellular distribution of glycogen in conidia and appressoria was observed at 0–24 (h) The staining of the samples with I_2_/KI solution showed a dark brown color of glycogen under a white light microscope. Scale Bar = 10 μm. **(D)** The number of conidia containing glycogen as a percentage. **(E)** Cellular distribution of lipid droplets in conidia and appressoria was observed at 0–24 (h) Samples were stained with BODIPY solution, and lipid droplets displayed green fluorescence under a fluorescence microscope. Scale Bar = 10 μm. **(F)** The number of conidia containing lipid droplets as a percentage. **(G)** The number of appressoria containing lipid droplets as a percentage. Error bars represent the standard deviation. An analysis of the data was carried out using an unpaired two-tailed Student’s t-test. Asterisks represent statistically significant differences in the data (**P < 0.01, *P < 0.05).

Huge turgor pressure needs to accumulate in the appressorium to complete the penetration of plant epidermal cells (Howard and Valent, 1996; deJong et al., 1997). Therefore, we measured the turgor pressure of mature appressoria using incipient cytorrhysis assays. Following treatment with 1.0 M glycerol, the collapse rates of the wild-type and complementation strain appressoria reached nearly 20%, while those of Δ*Mosec24B* reached 50%. As glycerol concentration increased to 1.5 M, 70% of mature appressoria of *ΔMosec24B* collapsed, whereas only 20% of WT and Δ*Mosec24B::MoSEC24B* strains collapsed (**Figures 5A, B**). These results indicate that appressorium turgor is reduced significantly in Δ*Mosec24B*.

The synthesis of glycerol in the appressorium originates from the metabolism of glycogen and lipid droplets (Wang et al., 2007; Foster et al., 2017). To observe the turnover of glycogen and lipid droplets, I_2_/KI solution was used to stain glycogen and Bodipy was used to stain lipid droplets. Both the wild-type strain and the mutant conidia exhibited the presence of glycogen at 0 h as detected by I_2_/KI staining. From 4 h to 8 h, glycogen in conidia was transported to appressoria. From 16 h to 24 h, glycogen in the conidia continued to transport to appressoria, and glycogen in the appressoria was degraded at the same time. We observed that there was no difference in glycogen degradation between the wild-type and the mutant strain appressoria (**Figure S4**), but at 24 h, the glycogen in the wild-type and complementation strain conidia was completely transported to the appressoria. In contrast, nearly 15% of Δ*Mosec24B* conidia still contained glycogen (**Figures 5C, D**), indicating the delayed mobilization of glycogen in Δ*Mosec24B*. Similarly, for lipid droplet staining, we observed that the mobilization and degradation of lipid droplets were delayed in Δ*Mosec24B* (**Figures 5E–G**). These results indicate that MoSec24B impacts the translocation and degradation of glycogen and lipid droplets.

### Localization of MoRas1 is abnormal in the Δ*Mosec24B* mutant

**Figure 6:**
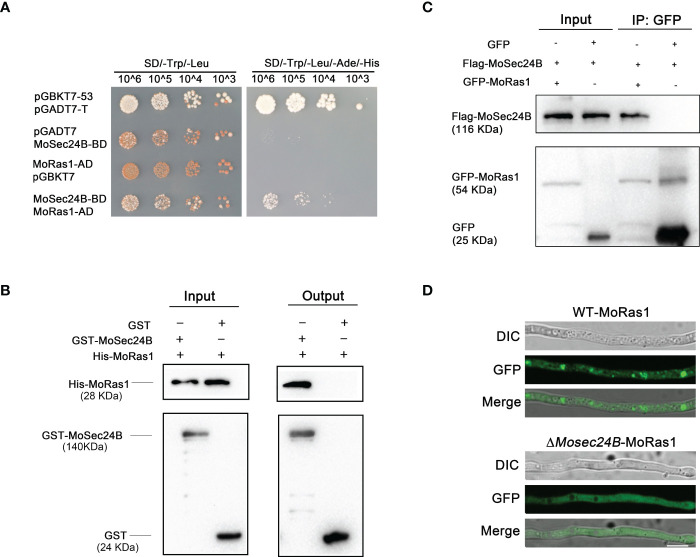
**(A)** Interaction between MoSec24B and MoRas1 was detected using a yeast-two-hybrid assay. pGBKT7-53 and pGADT7-T were used as positive controls, and pGBKT7 and pGADT7 were used as negative controls. **(B)** Interaction between MoSec24B and MoRas1 was examined using a pull-down assay. GST-MoSec24B and His-MoRas1, empty GST- and His-MoRas1 were incubated sequentially with glutathione agarose gel beads with GST labels for two hours, respectively. The final eluate obtained was detected by immunoprecipitation. **(C)** Interaction between MoSec24B and MoRas1 was detected using a co-IP assay. MoRas1-GFP and MoSec24B-Flag, empty GFP and MoSec24B-Flag were incubated with anti-GFP beads for four hours, respectively. The final eluate obtained was detected by immunoprecipitation. **(D)** Subcellular localization of MoRas1 in the wild-type and the Δ*Mosec24B* mutant mycelium. Scale Bar = 10 μm.

To further identify the pathways involved in the regulation of MoSec24B, we performed immunoprecipitation (IP) with MoSec24B-GFP as the bait protein and identified MoRas1 (Zhou et al., 2014; Aboelfotoh Hendy et al., 2019), a small GTPase capable of regulating the conversion of activated and inactivated GTP to GDP, in the proteins obtained by mass spectrometry (**Table S2**). We confirmed the interaction of MoSec24B with MoRas1 by yeast-two-hybrid, pull-down, and co-IP experiments (**Figures 6A–C**). Since the subcellular localization of Ras proteins is critical for their function (Zhou et al., 2014), we observed the localization of Ras1-GFP protein in the mycelium and found that the localization within the mutant was diffuse in the cytoplasm compare to WT (**Figure 6D**). These results suggest that the localization of MoRas1 is impaired in the Δ*Mosec24B* mutant.

### Localization of MoSep4 is abnormal in the Δ*Mosec24B* mutant

**Figure 7:**
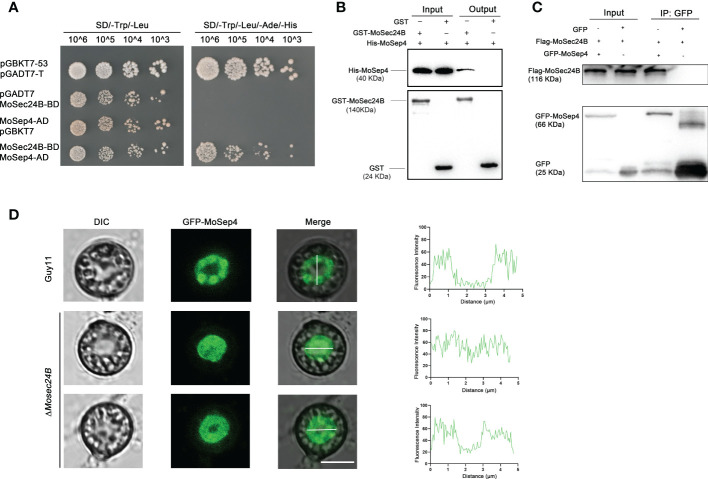
**(A)** Interaction between MoSec24B and MoSep4 was detected using a yeast-two-hybrid assay. **(B)** Interaction between MoSec24B and MoSep4 was examined using a pull-down assay. **(C)** Interaction between MoSec24B and MoSep4 was detected using a co-IP assay. **(D)** Subcellular localization of MoSep4 in the wild-type and Δ*Mosec24B* mutant appressoria (Fv3000, 60 × oil). Scale Bar = 5 μm.

When appressorium matures, it forms the septin ring structure at its base pore, and these ring structures participate in the infection process of *M. oryzae* by regulating the differentiation of the infection peg (Dagdas et al., 2012). Coincidently, we identified MoSep4, a core component of the septin complex, in the mass spectrometry results of IP (**Table S2**). We further confirmed the interaction between MoSec24B and MoSep4 by yeast-two-hybrid, pull-down and co-IP assays (**Figures 7A–C**). To further observe whether MoSec24B affects the ring structure of septins, we determined the localization of MoSep4-GFP in WT and Δ*Mosec24B*. The results showed that the wild-type appressoria showed a ring-like structure with a pore in the middle. However, the mutant formed two abnormal structures, one forming a uniformly dense structure without a pore in the middle and the other forming a ring-like structure with a narrow pore in the middle (**Figure 7D**). These results suggest that the ring structure of MoSep4 is abnormal in the Δ*Mosec24B* mutant.

### MoSec24B participates in the three MAPK kinase signaling pathways in *M. oryzae* through interaction with MoMst50

**Figure 8:**
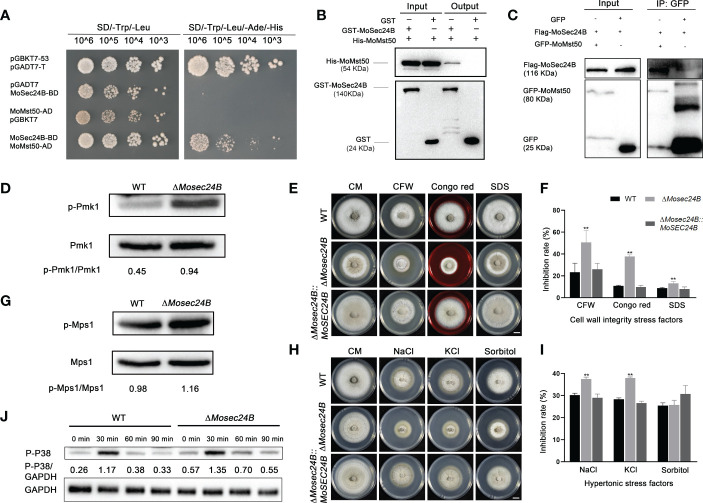
**(A)** Interaction between MoSec24B and MoMst50 was detected using a yeast-two-hybrid assay. **(B)** Interaction between MoSec24B and MoMst50 was examined using a pull-down assay. **(C)** Interaction between MoSec24B and MoMst50 was detected using a co-IP assay. **(D)** Phosphorylation analysis of Pmk1 in WT and Δ*Mosec24B*. **(E)** Mycelial plugs from WT, Δ*Mosec24B*, and Δ*Mosec24B::MoSEC24B* strains were grown on CM medium supplemented with 100 μg/ml CFW, 800 μg/ml CR, and 0.004% SDS for 8 days. Scale Bar = 1 cm. **(F)** The growth inhibition rates of WT, Δ*Mosec24B*, and Δ*Mosec24B::MoSEC24B* strains on CFW, CR, and SDS. Error bars represent the standard deviation. **(G)** Phosphorylation analysis of Mps1 in WT and Δ*Mosec24B* under treatment with 100 μg/ml CFW. **(H)** Mycelial plugs from WT, Δ*Mosec24B*, and Δ*Mosec24B::MoSEC24B* strains were grown on CM medium containing 0.6 M NaCl, 0.6 M KCl, and 1 M sorbitol for 8 days. Scale Bar = 1 cm. **(I)** The growth inhibition rates of WT, Δ*Mosec24B*, and Δ*Mosec24B::MoSEC24B* strains on NaCl, KCl, and sorbitol. Error bars represent the standard deviation. An analysis of the data was carried out using an unpaired two-tailed Student’s t-test. Asterisks represent statistically significant differences in the data (**P < 0.01). **(J)** Phosphorylation analysis of Osm1 in WT and Δ*Mosec24B* under treatment with NaCl using the protein content of GAPDH as a control.

MAPK cascade reaction plays an important role in the pathogenicity of *M. oryzae* (Park et al., 2006). We used the yeast-two-hybrid, pull-down, and co-IP methods to verify a direct interaction between MoSec24B and MoMst50, which is a bridging protein for the Mst11-Mst7 cascade reaction and activates the Pmk1-MAPK signaling pathway (**Figures 8A–C**). MoMst50 activates phosphorylation of Pmk1 (Li et al., 2017). Thus, we examined the phosphorylation level of Pmk1 in WT and Δ*Mosec24B* and found that the phosphorylation level of Pmk1 was significantly elevated in Δ*Mosec24B* (**Figure 8D**). MoMst50 also functions by activating Mps1 in response to cell wall stress (Li et al., 2017). To identify whether MoSec24B plays a role in the cell wall integrity of *M. oryzae*, we monitored the tolerance to different kinds of cell wall stress factors, including CFW, CR, and SDS, in Δ*Mosec24B* (**Figure 8E**). On CM supplemented with 100 μg/ml CFW, 800 μg/ml CR and 0.004% SDS, the growth inhibition rates of Δ*Mosec24B* were approximately 30%, 30% and 5% higher than those of WT and Δ*Mosec24B::MoSEC24B*, respectively (**Figure 8F**). Next, we examined the phosphorylation of Mps1 in WT and Δ*Mosec24B* under the condition of CFW and found that the phosphorylation of Mps1 was significantly elevated in Δ*Mosec24B* (**Figure 8G**). MoMst50 can synergistically activate the phosphorylation of Osm1 under hypertonic stress with the hybrid histidine kinase MoHik1 (Li et al., 2017). To further identify the role of MoSec24B in hypertonic stress, the growth inhibition rates of Δ*Mosec24B* on CM medium containing NaCl, KCl, and sorbitol were measured (**Figure 8H**). On CM containing 0.6 M NaCl and 0.6 M KCl, the growth inhibition rates of Δ*Mosec24B* were approximately 10% higher than those of WT and Δ*Mosec24B::MoSEC24B*. On CM supplemented with 1 M sorbitol, there were no significant differences between Δ*Mosec24* and WT (**Figure 8I**). To further investigate whether MoSec24B is involved in regulating the Osm1 signaling pathway, we examined the changes in phosphorylation levels of Osm1 in WT and the Δ*Mosec24B* mutant under NaCl induction conditions and found that the phosphorylation levels of Osm1 were low in the wild-type at the beginning, increased when induced for 30 min, and then started to decrease all the way down at 60 min and 90 min, but we found that the phosphorylation levels were higher at each time point in the Δ*Mosec24* mutant than in the wild-type (**Figure 8J**). These results reveal that MoSec24B can play an important role in the three MAPK signaling pathways together with MoMst50.

### MoSec24B is involved in the later phase of autophagosome clearance

**Figure 9:**
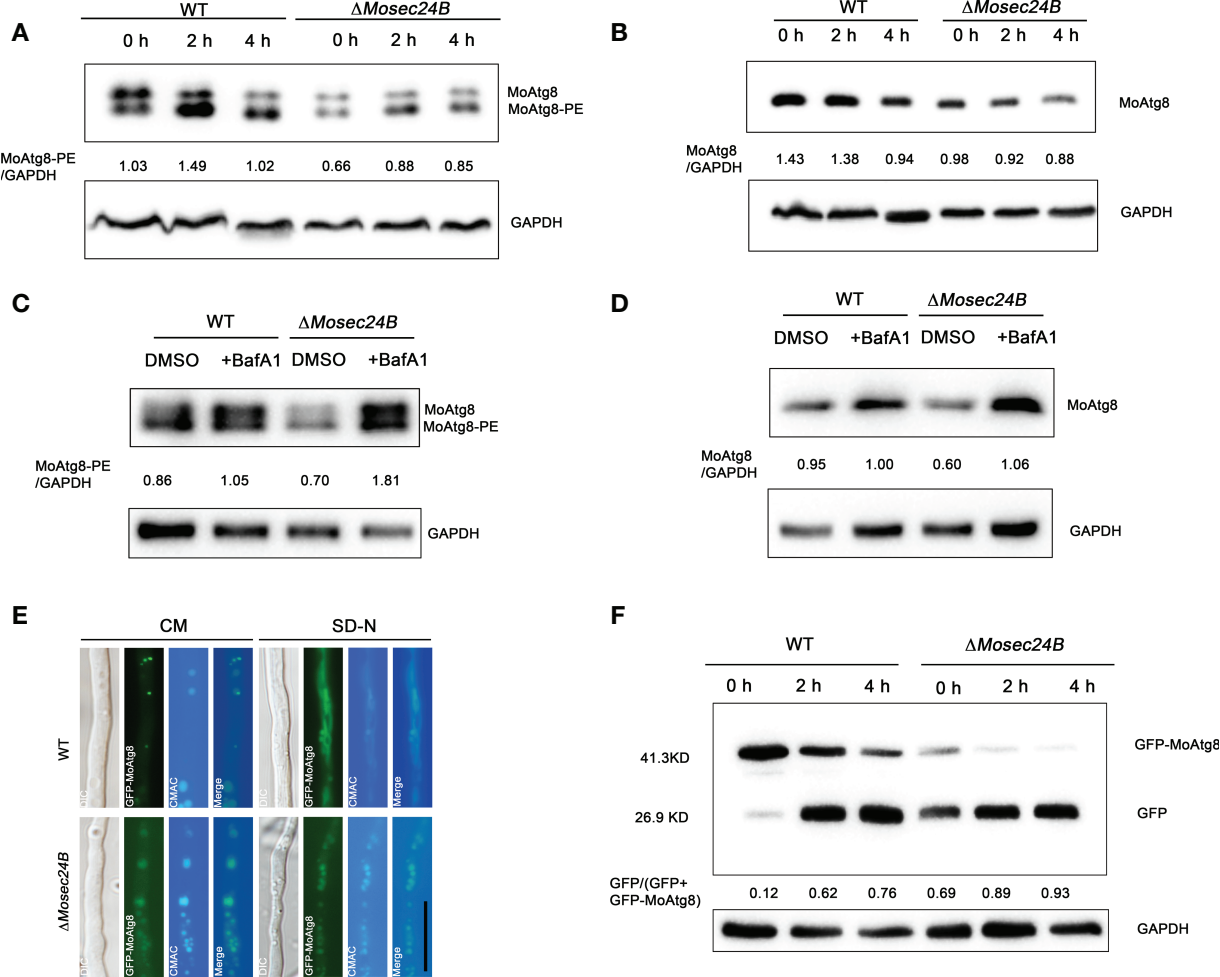
MoSec24B is engaged in the process of autophagy. **(A)** Analysis of MoAtg8 lipidation to form MoAtg8-PE in WT and Δ*Mosec24B*. To assess the conversion rate of MoAtg8 lipidation to form MoAtg8-PE, the ratio of total MoAtg8-PE to total GAPDH was calculated. **(B)** Analysis of MoAtg8 protein detection in WT and Δ*Mosec24B*. To assess the content of MoAtg8, the ratio of MoAtg8 to total GAPDH was calculated. **(C)** Analysis of MoAtg8 lipidation to form MoAtg8-PE in WT and Δ*Mosec24B* after BafA1 treatment for 4 h. **(D)** Analysis of MoAtg8 protein detection in WT and Δ*Mosec24B* after BafA1 treatment for 4 h. **(E)** Subcellular localization of GFP-MoAtg8 in WT and Δ*Mosec24B* under fluorescence microscopy. Scale Bar = 10 μm. Mycelia were stained with CMAC, which is used to stain vacuoles. **(F)** Autophagy flux analysis of GFP-MoAtg8 in WT and Δ*Mosec24B*. Total GFP-MoAtg8 and free GFP protein were detected by western blotting. The proportion of the amount of free GFP to the total amount of intact GFP-MoAtg8 and free GFP was calculated for the purpose of assessing the extent of autophagy. The protein content of GAPDH as a control. ImageJ software was used to analyze the grayscale value of each independent band.

Autophagy mediates the degradation of proteins or organelles in the face of cellular starvation, such as a lack of amino acids and sugar nutrients, and moderate autophagy contributes to energy storage (Liu et al., 2007; Lu et al., 2009; Yu et al., 2018). The Δ*Mosec24B* mutant showed a slowed translocation and degradation of glycogen and lipid droplets, and cellular autophagy in *M. oryzae* is closely linked to lipid metabolism. To identify whether MoSec24B functions in autophagy, we first assayed the conversion between Atg8 and Atg8-phosphatidylethanolamine (Atg8-PE) to assess the activity of autophagy. Atg8-PE is generally anchored to the autophagosome membrane and is a reliable autophagy reporter gene. And variations in Atg8-PE content have been reported to represent changes in the number of autophagosomes (Nair et al., 2012). The Δ*Mosec24B* mutant was shown to have consistently lower amounts of MoAtg8-PE than the wild-type at the three time points of nutrient enrichment and 2h, 4h induction by nitrogen starvation (**Figure 9A**). Furthermore, we examined the MoAtg8 content and found that the Δ*Mosec24B* mutant had less MoAtg8 than WT at any one time point (**Figure 9B**). Thus, we treated the mycelium induced by nitrogen starvation for 4 h with bafilomycin A1 (BafA1), an agent that inhibits the fusion of autophagosomes with vacuoles (Ammanathan et al., 2020), and found that the amount of MoAtg8-PE in the Δ*Mosec24B* mutant was more than that in the wild-type after using BafA1 (**Figure 9C**). Similarly, we also examined the content of endogenous Atg8 after the addition of BafA1 and found consistent results with those of Atg8-PE (**Figure 9D**). These results illustrate that MoSec24B affects the clearance of autophagosomes. Furthermore, we examined the degradation of GFP-MoAtg8, GFP-MoAtg8 fusion protein was transformed into WT and Δ*Mosec24B*. Autophagic flux was detected by observing the subcellular localization of GFP-MoAtg8 under a fluorescence microscope and analyzing free GFP via western blotting. In WT, GFP-MoAtg8 fluorescence appeared in the form of dots around the vacuoles in liquid CM. However, there was almost no dot structure in Δ*Mosec24B*. After the hyphae of strains were starved in SD-N liquid medium for 4 h, GFP-MoAtg8 fluorescence was present in the vacuole both in WT and the Δ*Mosec24B* mutant (**Figure 9E**). Apart from that, we also performed GFP-MoAtg8 proteolysis assays. At 0 h, the full-length GFP-MoAtg8 band was strong, and the free GFP band was weak in the wild-type strain, while the free GFP band was strong in Δ*Mosec24B*. After 4 h, the GFP-MoAtg8 band in Δ*Mosec24B* almost disappeared compared to WT (**Figure 9F**). These results indicate that fusion of autophagosomes with vacuoles was accelerated in Δ*Mosec24B.*

## Discussion

**Figure 10:**
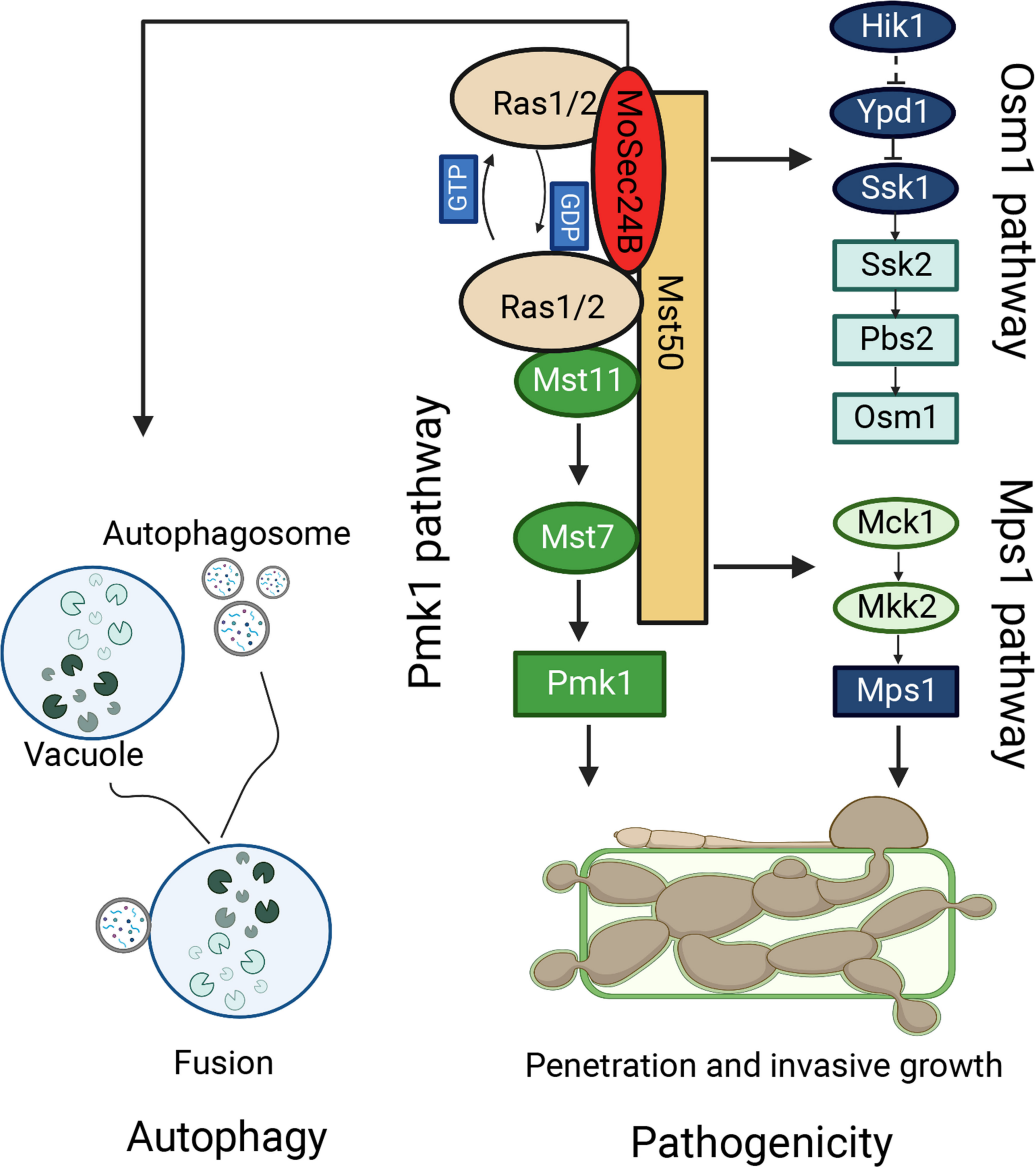
The model of MoSec24B in signaling pathways and autophagy. MoSec24B interacts with both MoRas1 and MoMst50, participating in the Pmk1, Mps1, and Osm1 pathways. Additionally, MoSec24B is involved in autophagy process, which affects the pathogenicity of *M. oryzae*. Autophagy, double membrane structure is autophagosome and single membrane structure is vacuole.

In pathogenic fungi, vesicle transport proteins can exert a major impact on fungal growth and the pathogenic process (Dou et al., 2011; Liu et al., 2021). Three main classes of vesicular proteins, Clathrin, COPI, and COPII, are present in eukaryotes (Barlowe and Miller, 2013). COPII, an ancient and conserved secretory pathway, mainly transports proteins and lipids to the Golgi for further protein processing and modification (Novick et al., 1980). Recent studies have shown that the transport process of COPII vesicles can facilitate the secretion of apoplastic effectors into rice, thereby regulating the pathogenicity of *M. oryzae* and the immune response of the host plant (Liu et al., 2021; Qian et al., 2022). However, it is not clear how COPII vesicles function in other modules to assist in the infection process of *M. oryzae* in addition to own secretion function. In this research, we identified basic biological functions of MoSec24B and discovered that the defects in fungal development and pathogenicity caused by disruption of MoSec24B are due to disruption of MAPK signaling and accelerated fusion of autophagosomes with vacuoles (**Figure 10**).

It has been well demonstrated that at least 122 kinases/phosphatases control the transport of the ER-Golgi secretory pathway by RNA interference screening and the MAPK signaling pathway can mediate the trafficking of ER to Golgi proteins and the generation of COPII vesicles (Farhan et al., 2010). Notably, we identified that MoSec24B could interact with MoRas1 and MoMst50. In *M. oryzae*, it was shown that MoRas1 only regulates conidial production and interacts with MoMst50 and MoMst11, key components of the Pmk1 pathway (Park et al., 2006). Furthermore, when the Ras-association domain in MoMst11 was knocked out, Pmk1 was not appropriately activated, and appressorium formation was abnormal (Qi et al., 2015), indicating that the normal signaling of Ras could activate the downstream Pmk1 pathway. MoMst50, as an adapter protein, can directly interact with MoMst11 and MoMst7, and then MoMst7 can interact with Pmk1 during appressorium formation period, which in turn activates the phosphorylation of Pmk1 (Li et al., 2017). When MoMst50, MoMst11, MoMst7, and MoPmk1 were knocked out separately in *M. oryzae*, it was found that spore production in these mutants was drastically reduced, the germ tubes were abnormally enlarged, and almost no phosphorylation of Pmk1, which could not form appressoria properly, thus reduce the ability to infect (Zhao et al., 2005; Zhou et al., 2014). Here, we found that the Δ*Mosec24B* mutant showed a dramatic decrease in spore production, normal appressorium formation but failure to maintain huge turgor pressure, and abnormally high level of Pmk1 phosphorylation, resulting in a significant reduction in the ability of appressorium-mediated infection. Moreover, previous studies reported that the phosphorylation level of Pmk1 was elevated in the Δ*Mocpka*Δ*Mocpk2* double knockout mutant and the Δ*MoPmp1* mutant, which resulted in reduced infection ability as well (Selvaraj et al., 2017; Wang et al., 2017). These results suggest that appressorium-mediated infection process can function normally only when the phosphorylation of Pmk1 is activated normally.

MoMst50 is involved in the regulation of the Mck1-Mkk2-mediated Mps1 pathway in addition to the Pmk1 signaling pathway. MoMst50 can interact with MoMck1 and MoMkk2, and then MoMck1 interacts with MoMkk2 to activate the phosphorylation of Mps1 (Li et al., 2017). The Mps1 pathway mainly regulates cell wall synthesis and participates in the appressorium-mediated infection process (Turra et al., 2014). The Δ*Momst50*, Δ*Momck1*, Δ*Momkk2*, Δ*Momps1* mutants all exhibited cell wall defects, reduced levels of Mps1 phosphorylation, and almost complete loss of pathogenicity to plants (Yin et al., 2016; Jiang et al., 2018). In our study, the growth of the Δ*Mosec24B* mutant was significantly inhibited on plates supplemented with the cell wall stress factors, including CFW, CR, and SDS, and the phosphorylation of Mps1 was abnormally elevated upon the addition of CFW compared to the wild type. Moreover, when MoMip11, a component of the Mps1 pathway, was knocked out, the mutant produced appressoria normally, sensitivity to cell wall stress factors and elevated phosphorylation levels of Mps1 (Li et al., 2017). We believe that the cell wall integrity of *M. oryzae* was disrupted after MoSec24B knockout, and the mutant enhanced the activation of Mps1 to mitigate this unfavorable situation and restore the normal function of the cell wall. However, the elevated phosphorylation level of Mps1 did not completely rescue the cell wall disruption, and we speculate that it may be due to the dramatic down-regulation of the expression of genes related to cell wall integrity. In addition, MoMst50 interacts with a filamentous fungus-specific osmotic stress sensor, MoMip7, which activates the Osm1 signaling pathway (Li et al., 2017). However, this pathway only regulates the magnitude of osmotic pressure in response to hypertonic stress. In our study, the Δ*Mosec24B* mutant was highly sensitive to ionic osmotic factors, growth was significantly inhibited in medium supplemented with NaCl and KCl, and the phosphorylation level of Osm1 was elevated. In summary, either enhanced or reduced phosphorylation of proteins on different MAPK signaling pathways, the appressorium could not function properly. Overall, we found that MoSec24B is involved in the three MAPK pathways in *M. oryzae* by interacting with MoRas1 and MoMst50, thereby regulating the process of plant infection.

Autophagy has been proven to play an important role in the pathogenicity of rice blast fungus in previous studies (Liu et al., 2007; Dong et al., 2009; Kershaw and Talbot, 2009; Lu et al., 2009). It was well known that when cells are induced by exogenous starvation, Sec24 in yeast can detach from the traditional secretory pathway of transporting cargo proteins from the ER to the Golgi and shift to participate in the autophagy pathway. During this period, upstream casein kinase 1 (Hrr25) is able to regulate the phosphorylation of three conserved sites on the distal surface of the Sec24 membrane. When Sec24 is phosphorylated, the interaction of Sec24 with Atg9, a key component that initiates autophagosome formation, is enhanced, thus promoting an increase in the number of autophagosomes (Davis et al., 2016). Here, we found that like the ATG-deficient mutant, Δ*Mosec24B* exhibited decreased conidiation, impaired appressorium turgor pressure, restricted glycogen and lipid droplets utilization, and lower pathogenicity to rice leaves (Veneault-Fourrey et al., 2006; Liu et al., 2007). Moreover, the content of Atg8-PE in the wild type and Δ*Mosec24B* mutant was examined in our study, and we found that the Δ*Mosec24B* mutant had less Atg8-PE than the wild type under normal CM liquid culture as well as SD-N medium. Two possible reasons for the decrease in Atg8-PE content could exist, one being the low content of autophagosomes formed by autophagy induction itself, and the other being the accelerated fusion of autophagosomes with vacuoles. However, the content of Atg8-PE in the Δ*Mosec24B* mutant was more than wild type after the addition of BafA1. Furthermore, we also examined the degradation of GFP-MoAtg8 and found that the degradation of GFP-MoAtg8 was accelerated in Δ*Mosec24B*, indicating that the fusion of late autophagosomes with vacuoles was accelerated in Δ*Mosec24B*. Based on the above results, we believe that the resulting accelerated autophagy flux in Δ*Mosec24B* resulted in reduced appressorium turgor and attenuated plant infection. Additionally, MoSec24B has been reported to interact with MoVps27, a component of endosomal sorting complexes required for transport-0 (ESCRT-0). Studies have found that the Δ*Movps27* mutant led to no conidiation, complete loss of pathogenicity, and enhanced autophagy flux (Sun et al., 2022). Moreover, previous studies have shown that the CWI pathway is vital for governing in response to the external environment in fungi (Levin, 2005; Li et al., 2012a). Autophagy and CWI signals synergistically govern the pathogenicity of *M. oryzae*. Under DTT stress, the autophagy core protein MoAtg1 can phosphorylate MoMkk1, thereby activating the CWI pathway and regulating the pathogenicity of the rice blast fungus (Yin et al., 2020). This sheds new light on the next step of our study. Exploring how MoSec24B functions in CWI and autophagy pathways will help to investigate the pathogenic mechanism of *M. oryzae* and thus control the break-out of this devastating disease.

In conclusion, we reveal that MoSec24B plays an important role in the growth and pathogenicity of *M. oryzae*. And, we put forward MoSec24B to participate in three MAPK signaling pathways and the late fusion process of autophagosomes and vacuoles to regulate the pathogenicity of the rice blast fungus.

## Data availability statement

The datasets presented in this study can be found in online repositories. The names of the repository/repositories and accession number(s) can be found in the article/**Supplementary Material**.

## Author contributions

XL and HQ designed the experiment and wrote the manuscript. LS, MW, ML, WZ, SL, XZ, and LL performed data analysis and scripts. FL, XL, JL, and ZS provided experimental materials and technical support. All authors contributed to the article and approved the submitted version.

## Supplementary material

Supplementary Figure 1 Identification of Δ*Mosec24B*. **(A)** Schematic diagram of knockout by homologous recombination. **(B)** A band of 1.2-1.5 kb from the null mutants was amplified but not from WT. PCR screening of the obtained gene deletion mutants using a single copy of the β-tubulin gene as a positive control. WT can be detected with a characteristic band, which is the targeted gene, whereas the null mutants cannot be detected. **(C)** Verification of the insertion copy number of WT and the null mutant.

Supplementary Figure 2 Transcript expression of conidia-related genes in the Δ*Mosec24B* mutant aerial mycelium relative to the wild-type. Error bars represent the standard deviation. An analysis of the data was carried out using an unpaired two-tailed Student’s t-test. Asterisks represent statistically significant differences in the data (**P < 0.01).

Supplementary Figure 3 The appressorium formation experiment of Δ*Mosec24B*. **(A)** The appressoria of WT, Δ*Mosec24B*, and Δ*Mosec24B::MoSEC24B* strains at 24 h. Scale Bar = 10 μm. **(B)** Conidial germination rates of WT, Δ*Mosec24B*, and Δ*Mosec24B::MoSEC24B* strains at 4 h. **(C)** Appressorium formation rates of WT, Δ*Mosec24B*, and Δ*Mosec24B::MoSEC24B* strains at 8 h and 24 h. Error bars represent the standard deviation. An analysis of the data was carried out using an unpaired two-tailed Student’s t-test.

Supplementary Figure 4 The percentage of appressoria containing glycogen. Error bars represent the standard deviation. An analysis of the data was carried out using an unpaired two-tailed Student’s t-test.
