# Supplementary material for: The COPII subunit MoSec24B is involved in development, pathogenicity and autophagy in the rice blast fungus
Source: Front Plant Sci. 2023 Jan 9;13:1074107. doi: 10.3389/fpls.2022.1074107 (PMC9868959; doi:10.3389/fpls.2022.1074107)
Supplement: Supplementary file 1 [file Table_1.docx]

**Table S1** Primers used in this study

| **Primer name** | **Primer sequence (5’-3’)** |
| --- | --- |
| **Primers used for gene knock out** | |
| MoSec24B-UP-F | GGTACCCGGGGATCCTCTAGAGCTGTTGTCCATCCACTC |
| MoSec24B-UP-R | TTCATTGTTGACCTCCACTATCTTGCCTTCAGGTGCTA |
| MoSec24B-Down-F | GCAAAGGAATAGAGTAGATGTCTCCGATGTGTATCAGTTC |
| MoSec24B-Down-R | ACGGCCAGTGCCAAGCTTTGCCATTGCTTCTTGTGT |
| HPH-F | TAGTGGAGGTCAACAATGAATG |
| HPH-R | CATCTACTCTATTCCTTTGCCC |
| MoSec24B-L-F | GGTGTCGGTAGCCAATATG |
| HPH-CK-R | GATAATAATGTCCTCGTTCC |
| MoSec24B-SF | GTCAGCGTATGTTATCTCTTC |
| MoSec24B-SR | GTTGCGATGTCCAGGTAG |
| TUBLIN-F | CCATCCCGAGCTTGTTGATA |
| TUBLIN-R | GTAGTTCAGGTCACCGTATGAG |
| **Primers** **used for fluorescent observation** | |
| MoSec24B-mCherry-F | CCACCGGCGGCTAATCTAGAATGGCCGACTACTCTATGTA |
| MoSec24B-mCherry-R | TACTGCAGGTCGACTCTAGACCAGTACCCAGGTCGCAAAC |
| MoSec24A-GFP-F | CAATCACAATGGCCGGATCCATGTCAGCCCCAGACCAAGG |
| MoSec24A-GFP-R | CCCTTGCTCACCATCCCGGGCTGAACTACCTGGAATACCA |
| MoSlp1-GFP-F | CAACCGTCAAAATGGTCGGCATACGCCGAAACTTCATAGA |
| MoSlp1-GFP-R | CCTCGCCCTTGCTCACCATCCTTGCGGCAGCAGGCTGT |
| MoSft2-GFP-F | CCGTCAAAATGGTCGGATCCATGGCATCGCAGTCATTCCG |
| MoSft2-GFP-R | CCCTTGCTCACCATCCCGGGGCCAGTCATCCAAGCGGCCG |
| MoSep4-GFP-F | CAATCACAATGGCCGGATCCATGGCGGCCATGCCTGCTGC |
| MoSep4-GFP-R | CCCTTGCTCACCATCCCGGGGTAGCCATTCATAGTCATCC |
| MoRas1-GFP-F | CAATCACAATGGCCGGATCCATGACTGGAAGGTTGCAGCT |
| MoRas1-GFP-R | CCCTTGCTCACCATCCCGGGCAATATAACACACTTGATCG |
| **Primers used for Yeast-two-hybrid** | |
| MoSec24A-AD-F | GGAGGCCAGTGAATTCATGTCAGCCCCAGACCAAGGTT |
| MoSec24A-AD-R | CGAGCTCGATGGATCCCTGAACTACCTTCTCCCTC |
| MoSec24B-BD-F | CATGGAGGCCGAATTCATGGCCGACTACTCTATGTAT |
| MoSec24B-BD-R | GCAGGTCGACGGATCCCCAGTACCCAGGTCGCAAAC |
| MoSec23-AD-F | GGAGGCCAGTGAATTCATGGATTATGAAAACCTGAAGG |
| MoSec23-AD-R | CGAGCTCGATGGATCCGTTGGTGCCACTAACAGCCAACT |
| MoSec23-BD-F | CATGGAGGCCGAATTCATGGATTATGAAAACCTGAAGG |
| MoSec23-BD-R | GCAGGTCGACGGATCCGTTGGTGCCACTAACAGCCAACT |
| MoSec13-BD-F | CATGGAGGCCGAATTCATGATTCACGACGCAGTTCT |
| MoSec13-BD-R | GCAGGTCGACGGATCCGTCCTCGAGGCTGTTGACGCAAG |
| MoSec31-AD-F | GGAGGCCAGTGAATTCATGGTCCGACTTAGGGAGATC |
| MoSec31-AD-R | CGAGCTCGATGGATCCAGGAGTAGCCTTGCTCAT |
| MoRas1-AD-F | GGAGGCCAGTGAATTCATGACTGGAAGGTTGCAGC |
| MoRas1-AD-R | CGAGCTCGATGGATCCCAATATAACACACTTGATCGGC |
| MoSep4-AD-F | GGAGGCCAGTGAATTCATGGCGGCCATGCCTGCT |
| MoSep4-AD-R | CGAGCTCGATGGATCCGTAGCCATTCATAGTCATCCTT |
| MoMst50-AD-F | GGAGGCCAGTGAATTCATGAGCTTCAACACGGGGACGG |
| MoMst50-AD-R | CGAGCTCGATGGATCCTATTATTCCTCCTGGGGGAT |
| **Primers used for Pull-down** | |
| GST-MoSec24B-F | CCGCGTGGATCCCCGGAATTCATGGCCGACTACTCTATG |
| GST-MoSec24B-R | CTCGAGTCGACCCGGGAATTCCCAGTACCCAGGTCGCAAA |
| His-MoRas1-F | CGAGCTCCGTCGACAAGCTTATGACTGGAAGGTTGCAGC |
| His-MoRas1-R | TCGAGTGCGGCCGCAAGCTTCAATATAACACACTTGATCG |
| His-MoSep4-F | CGAGCTCCGTCGACAAGCTTATGGCGGCCATGCCTGCT |
| His-MoSep4-R | TCGAGTGCGGCCGCAAGCTTGTAGCCATTCATAGTCATCCTT |
| His-MoMst50-F | CAAGGTCGACAAGCTTATGAGCTTCAACACGGGGACGG |
| His-MoMst50-R | GTGCGGCCGCAAGCTTTATTATTCCTCCTGGGGGAT |
| **Primers used for co-IP** | |
| MoMst50-GFP-F | CAATCACAATGGCCGGATCCATGAGCTTCAACACGGGGAC |
| MoMst50-GFP-R | CCCTTGCTCACCATCCCGGGTATTATTCCTCCTGGGGGAT |
| MoSec24B-Flag-F | CAATCACAATGGCCGGATCCATGGCCGACTACTCTATGTA |
| MoSec24B-Flag-R | TGGTCCTTGTAGTCCCCGGGCCAGTACCCAGGTCGCAAAC |
| **Primers used for qPCR** | |
| RT-HPH-F | ATGTCCTGCGGGTAAATAGC |
| RT-HPH-R | GATGCAATAGGTCAGGCTCTC |
| RT-TUBLIN-F | ACAACTTCGTCTTCGGTCAG |
| RT-TUBLIN-R | GTGATCTGGAAACCCTGGAG |
| RT-HOX2-F | GGACTGCCTCTCAATGGTATG |
| RT-HOX2-R | AGGTGTGGGAGCAATTATCG |
| RT-CoNx2-F | GCCCGAGAGCAAGAGTAATAC |
| RT-CoNx2-R | CTGCTTCATGTACGGGTAGTC |
| RT-FLBC-F | TCCCTCAACTACGACCCTTAC |
| RT-FLBC-R | GTGAGCTGCCAGAGTAGATAC |
| RT-CNF1-F | ACCTTTCGACTACCGCAAC |
| RT-CNF1-R | CAGTTCATACATTTGCCATCGG |
| RT-GTA1-F | AAGACATCCACGACCAGTTC |
| RT-GTA1-R | GTAACGTGGTTCCTGGTAGC |
| RT-MSTU1-F | GATCAGGGTCACAATAACGGG |
| RT-MSTU1-R | ACTAGAGATGGACGAGACAGG |
| RT-Actin-F | ACAATGGTTCGGGTATGTGC |
| RT-Actin-R | CGACAATGGACGGGAAGAC |
| RT-40S-F | ACAAGCTCAAGACCCTCGTC |
| RT-40S-R | GGTGGTGATGGTGAAGCAG |
