# Supplementary material for: The COPII subunit MoSec24B is involved in development, pathogenicity and autophagy in the rice blast fungus
Source: Front Plant Sci. 2023 Jan 9;13:1074107. doi: 10.3389/fpls.2022.1074107 (PMC9868959; doi:10.3389/fpls.2022.1074107)
Supplement: Supplementary file 2 [file Table_2.docx]

**Table S2** Proteins identified by mass spectrometry that interacted with MoSec24B.

| **Gene ID/name** | **MW [kDa]** | **Score** | **Unique Peptides** |
| --- | --- | --- | --- |
| MGG_06910/Sec23 | 85.9 | 2225.21 | 22 |
| MGG_06726/Sep4 | 38.7 | 30.79 | 1 |
| MGG_09499/Ras1 | 26.9 | 0 | 1 |
