# Supplementary figures and images for: The COPII subunit MoSec24B is involved in development, pathogenicity and autophagy in the rice blast fungus
Source: Front Plant Sci. 2023 Jan 9;13:1074107. doi: 10.3389/fpls.2022.1074107 (PMC9868959; doi:10.3389/fpls.2022.1074107)

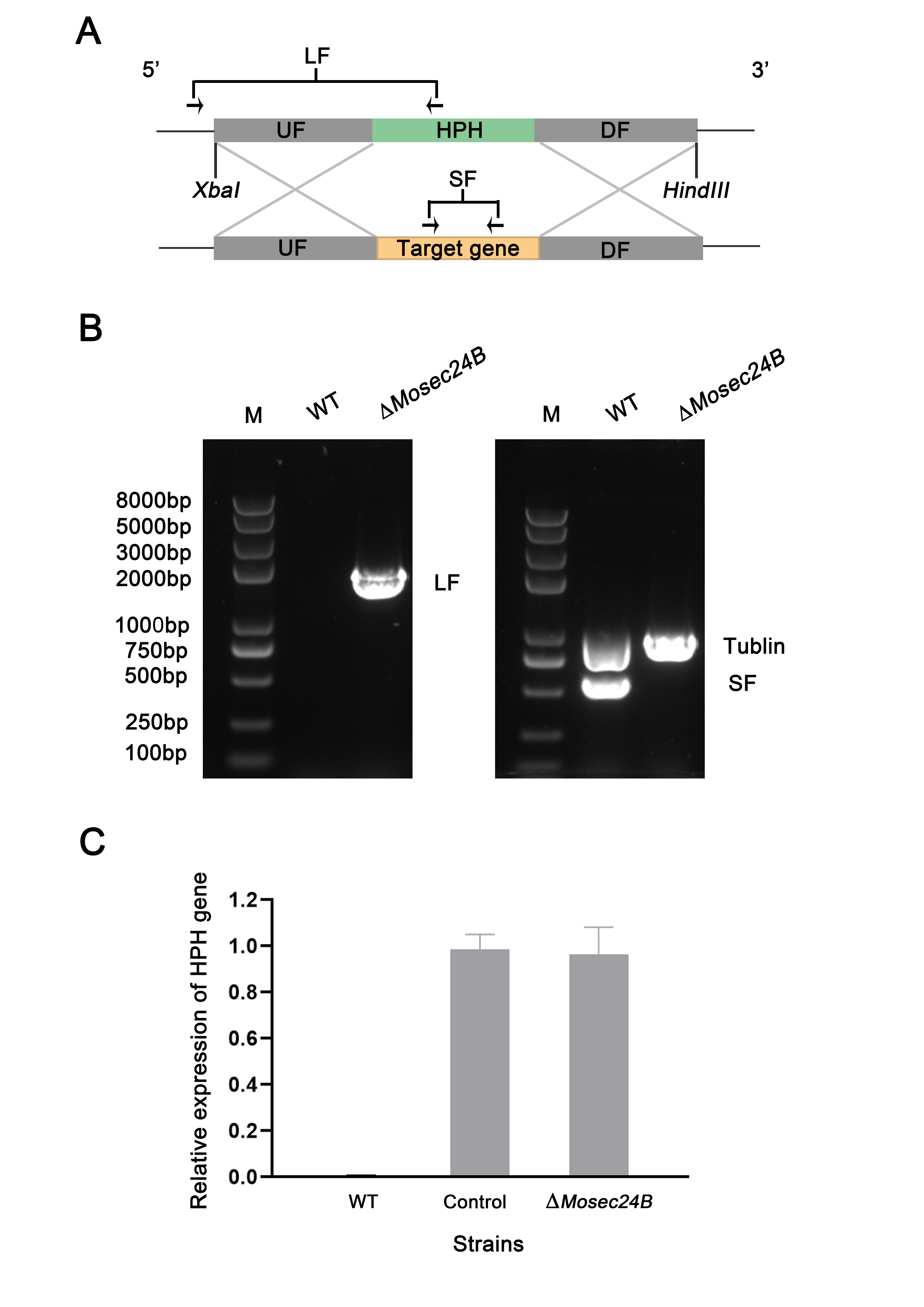

Supplement: Supplementary Figure 1 — Identification of ΔMosec24B. (A) Schematic diagram of knockout by homologous recombination. (B) A band of 1.2-1.5 kb from the null mutants was amplified but not from WT. PCR screening of the obtained gene deletion mutants using a single copy of the β-tubulin gene as a positive control. WT can be detected with a characteristic band, which is the targeted gene, whereas the null mutants cannot be detected. (C) Verification of the insertion copy number of WT and the null mutant. [file Image_1.tif]

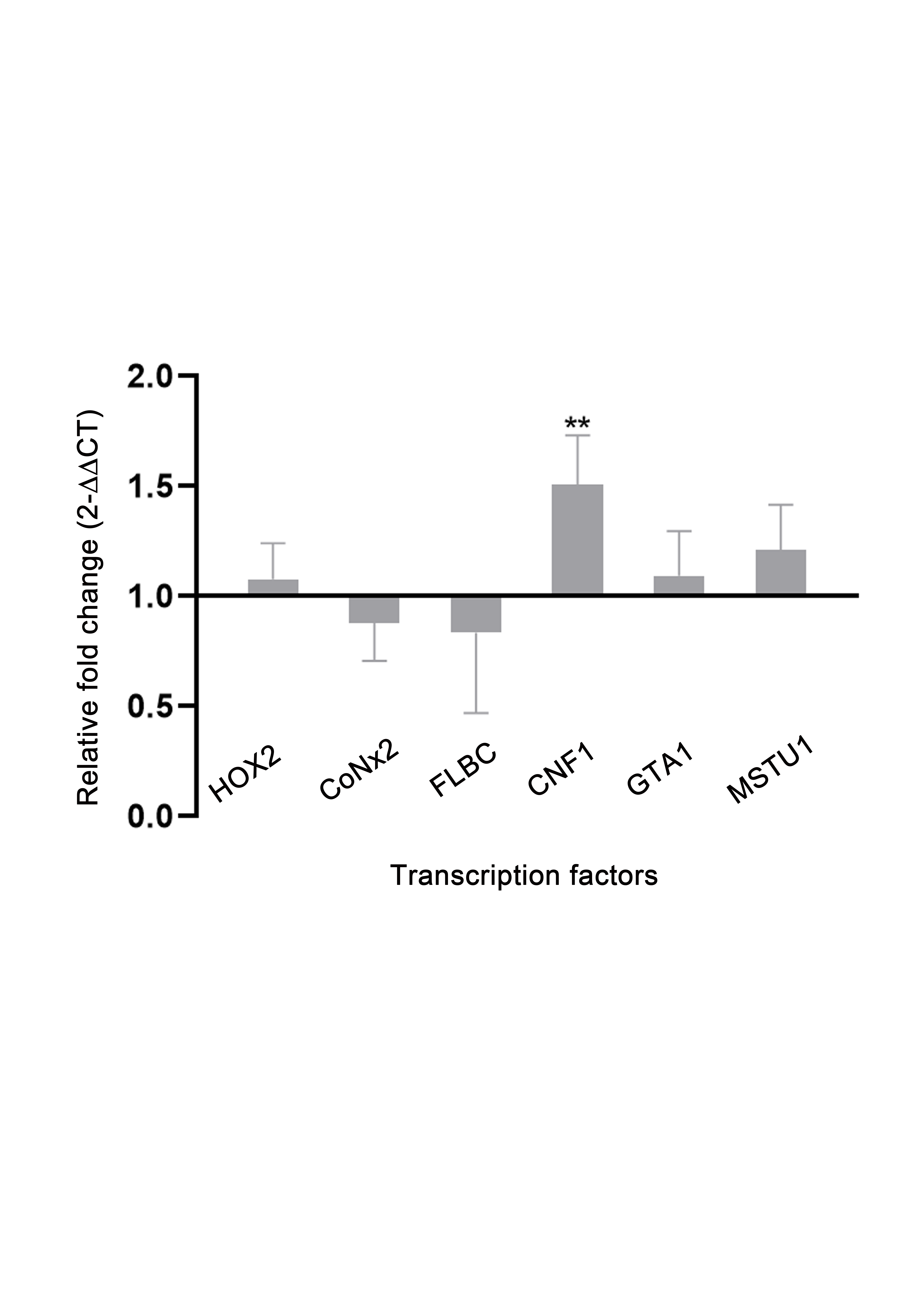

Supplement: Supplementary Figure 2 — Transcript expression of conidia-related genes in the ΔMosec24B mutant aerial mycelium relative to the wild-type. Error bars represent the standard deviation. An analysis of the data was carried out using an unpaired two-tailed Student’s t-test. Asterisks represent statistically significant differences in the data (**P < 0.01). [file Image_2.tif]

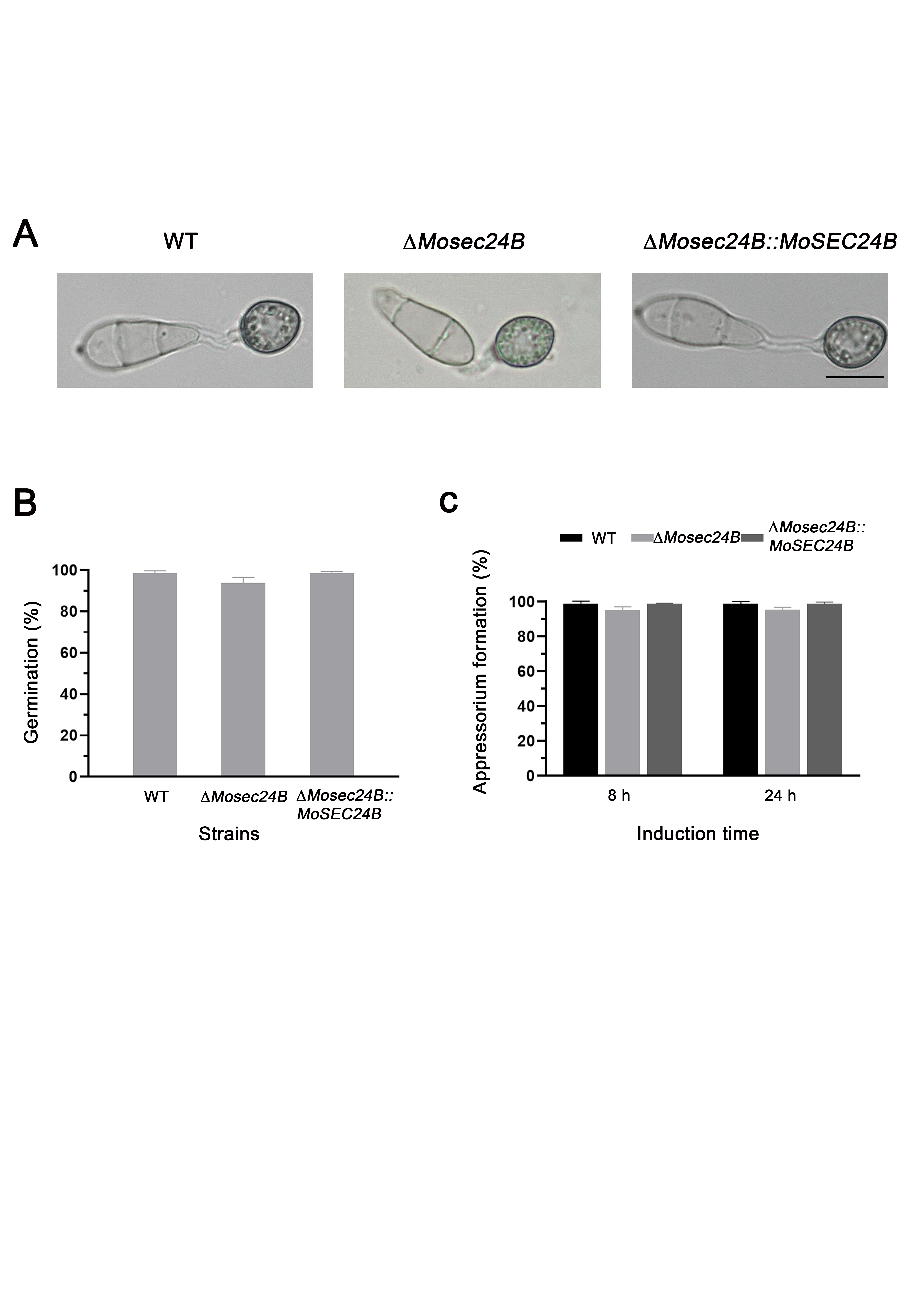

Supplement: Supplementary Figure 3 — The appressorium formation experiment of ΔMosec24B. (A) The appressoria of WT, ΔMosec24B, and ΔMosec24B::MoSEC24B strains at 24 h. Scale Bar = 10 μm. (B) Conidial germination rates of WT, ΔMosec24B, and ΔMosec24B::MoSEC24B strains at 4 h. (C) Appressorium formation rates of WT, ΔMosec24B, and ΔMosec24B::MoSEC24B strains at 8 h and 24 h. Error bars represent the standard deviation. An analysis of the data was carried out using an unpaired two-tailed Student’s t-test. [file Image_3.tif]

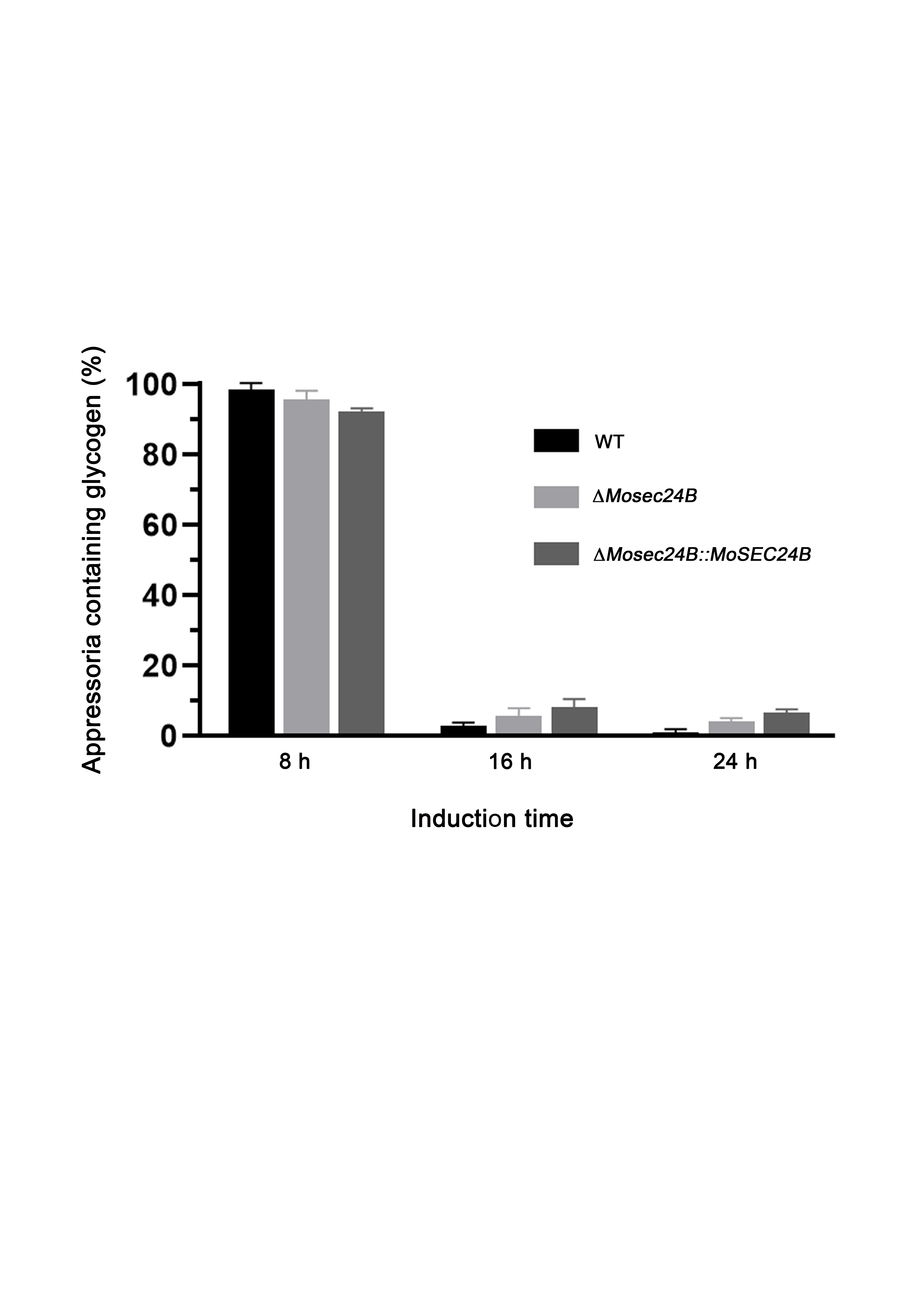

Supplement: Supplementary Figure 4 — The percentage of appressoria containing glycogen. Error bars represent the standard deviation. An analysis of the data was carried out using an unpaired two-tailed Student’s t-test. [file Image_4.tif]
